# Evaluation of Intra-Amniotic Infection Detection Based on Available Diagnostic Methods

**DOI:** 10.3390/medicina61122227

**Published:** 2025-12-17

**Authors:** Magda Nawceniak-Balczerska, Andrzej Torbé, Piotr Tousty, Olimpia Sipak-Szmigiel, Aneta Cymbaluk-Płoska, Justyna Kordek, Krzysztof Kaczmarek, Agnieszka Kordek

**Affiliations:** 1 Department of Obstetrics and Gynecology, Multi-Specialist Provincial Hospital in Gorzów Wlkp., 66-400 Gorzów Wielkopolski, Poland; 2 Department of Obstetrics and Gynecology, Pomeranian Medical University in Szczecin, 70-111 Szczecin, Poland; 3 Department of Obstretrics and Gynecology, University Clinical Hospital No. 2 in Szczecin, 70-111 Szczecin, Poland; 4 Department of Reconstructive and Oncological Gynecology, Independent Public Specialized Health Care Facility “ZDROJE”, 70-780 Szczecin, Poland; 5 Regional Center of Blood Donation and Treatment, 70-482 Szczecin, Poland; 6 Department of Neonatology and Intensive Neonatal Care, Pomeranian Medical University in Szczecin, 72-010 Szczecin, Poland; agnieszka.kordek@pum.edu.pl

**Keywords:** C-reactive protein, intraamniotic infection, chorioamnionitis, interleukin-6, procalcitonin, glucose, early-onset neonatal infection, cervical canal swab

## Abstract

*Background and Objectives*: Despite the development of medicine, there is no clearly established scheme for the prediction of intra-amniotic infection (IAI). In this study, evaluation of some predictors of IAI confirmed in histopathological examination was performed. *Materials and Methods*: The study population included 70 patients all giving birth by cesarean section divided into two groups: study (*n* = 34) consisting of patients with histologically confirmed IAI and control (*n* = 36) without IAI. Biological material included the mother’s venous blood to determine C-reactive protein (CRP), interleukin-6 (IL-6) and procalcitonin (PCT) concentrations; vaginal discharge to determine IL-6; cervical canal swabs to perform cultures for bacteria and fungi and polymerase chain reaction (PCR) for *Ureaplasma urealyticum*, *Mycoplasma hominis*, and *Chlamydia trachomatis*; amniotic fluid to perform cultures for aerobic and anaerobic bacteria and PCR for atypical pathogens, and to determine glucose, IL-6, and PCT concentrations; umbilical cord blood to determine PCT, CRP, Il-6 and blood culture. A fragment of the placenta and fetal membranes was taken for histopathological assessment of the inflammatory infiltrate. *Results*: Mothers’ serum CRP assessments as well as serum PCT assessments are of poor diagnostic value in the prediction of IAI confirmed in histopathological examination. *Conclusions*: The best predictive values of IAI confirmed in histopathological examination were amniotic fluid glucose and vaginal fluid IL-6 determinations.

## 1. Introduction

Despite the enormous development of medicine, modern obstetrics still struggles with many clinical problems. One of them is intraamniotic infection (IAI), which is a serious problem in perinatology with huge medical and socio-economic effects on the population [1]. Intrauterine infection often results in preterm birth and, subsequently, complications of prematurity: bronchopulmonary dysplasia, intracranial hemorrhage, respiratory distress syndrome, necrotizing enterocolitis, retinopathy of prematurity, cerebral palsy and delayed psychomotor development [2]. The long-term consequences of prematurity constitute a significant problem in the economic, sociological and psychological dimensions. Prematurely born children require multi-specialist medical care and support for psychomotor development for many years [3,4].

Serious complications that may occur in pregnant women with IAI include sepsis, septic shock, puerperal infections, postpartum hemorrhage, termination of pregnancy by cesarean section due to abnormal labor function, postoperative wound infection, endometrial infection after delivery, respiratory distress syndrome, and coagulopathies. A cesarean section performed in a patient with intra-amniotic infection is associated with an increased risk of severe anemia with the need for blood transfusion, pelvic abscess formation, and septic thrombophlebitis of the pelvic veins. Despite the development of modern medicine, there is no clearly established scheme for the diagnosis, therapy and prevention of intra-amniotic infection [5].

Intra-amniotic infection can be recognized based on the presence of clinical symptoms, laboratory and microbiological test results, or histopathological evaluation of the placenta. Clinical chorioamnionitis was diagnosed by Gibbs et al. when maternal temperature was elevated (>38 °C), and two or more of the following criteria were presented: maternal (>100 beats/min) or fetal (>160 beats/min) tachycardia, uterine tenderness, foul-smelling amniotic fluid, elevated white blood cell (WBC > 15,000/mm^3^) count, and maternal plasma C-reactive protein (CRP) > 10 mg/L [6].

Recently, the term “intrauterine inflammation or infection or both” abbreviated as “Triple I” was suggested by a National Institute of Child Health and Human Development (NICHD) expert panel including maternal and neonatal experts in a workshop to replace the term chorioamnionitis [7,8].

In order to diagnose intra-amniotic infection, laboratory test results seem to be helpful, including white blood cell (WBC) levels, CRP, procalcitonin (PCT), proinflammatory cytokine concentrations and their increase in control tests [9]. For the diagnosis of histological chorioamnionitis, microscopic analysis of the placenta at 400× magnification and previously described diagnostic criteria of chorioamnionitis (>10 polymorphonuclear leucocytes in 10 non-adjacent microscopic fields from the extraplacental membranes, chorionic plate or umbilical cord blood vessels) were applied [10].

In some clinical centers, amniocentesis is routinely performed in cases of suspected intra-amniotic infection in patients with threatened preterm labor and preterm premature rupture of membranes (pPROM). The amniotic fluid glucose, lactate dehydrogenase, and leukocyte levels are then assessed, and microbiological cultures are performed. Diagnosis of intrauterine infection based on the examination of amniotic fluid collected during amniocentesis allows for the implementation of treatment that will ultimately improve the prognosis of newborns. However, this invasive procedure carries a certain risk of complications (infections, premature birth, spontaneous abortion, amniotic fluid leakage) [11].

In this study, evaluation of some predictors of intra-amniotic infection confirmed retrospectively in histopathological examination was performed. The aim of this study is to improve the effectiveness of diagnosing intra-amniotic infection. Early diagnosis of intra-uterine infection may improve the diagnostic process for newborns and enable faster treatment implementation, which may result in reduced perinatal mortality and neonatal sepsis-related complications.

## 2. Materials and Methods

Patient population: The prospective study was conducted at the Department of Obstetrics and Gynecology of the Pomeranian Medical University in Szczecin (tertiary care referral center) with the consent of the Bioethics Committee (KB-0012/140/18), approval date: 26 December 2018.

The study population included 70 patients all giving birth by cesarean section. The patients expressed their informed consent to participate in the study after having read the information card describing the method of obtaining the research material.

Patients whose pregnancies were terminated by cesarean section were randomly qualified to participate in the study. The division into groups took place after obtaining the results of histopathological tests, which were considered to confirm and verify the presence of intrauterine infection.

Patients were divided into two groups: study (HY, *n* = 34) consisting of patients with histological confirmed intra-amniotic infection and control (HN; *n* = 36) without intra-amniotic infection.

Exclusion criteria: age below 18 years, lack of informed consent, vaginal delivery, and lack of complete data for analysis.

In order to assess the consistency of the diagnosis of intra-amniotic infection in the parturient with the diagnosis of early-onset infection (EONI) in the newborn, an analysis of the newborn’s medical records was performed, taking into account the clinical condition, use of antibiotic therapy and length of hospitalization.

On the day of delivery by cesarean section, the following samples were collected:

- Venous blood from the patient to determine CRP, IL-6, and PCT concentrations.
- Vaginal discharge to determine IL-6.
- Cervical canal swab to perform cultures for aerobic and anaerobic bacteria, atypical bacteria (*Ureaplasma* spp., *Mycoplasma hominis*, *Chlamydia trachomatis*) and fungi, and polymerase chain reaction (PCR) for *Ureaplasma urealyticum*, *Mycoplasma hominis*, and *Chlamydia trachomatis*.
- Amniotic fluid to perform cultures for aerobic and anaerobic bacteria and PCR for atypical pathogens (*Ureaplasma urealyticum*, *Mycoplasma hominis*, *Chlamydia trachomatis*), and to determine glucose, IL-6, and PCT concentrations.
- A fragment of the placenta and fetal membranes for histopathological assessment of the inflammatory infiltrate.
- Umbilical cord blood (5 mL) to determine PCT, CRP, and Il-6, and perform blood culture.

Methods: The concentration of CRP in peripheral blood was assessed using a test based on the COBAS INTEGRA 800 C-Reactive Protein (Latex CRPLX) cassette (ROCHE Diagnostic Systems Inc., Branchburg, NJ, USA). The electrochemiluminescence method using the COBAS 801 cassette was used to assess the concentrations of interleukin-6 and procalcitonin. Vaginal secretions were collected into sterile Eppendorf centrifuge tubes during a gynecological examination using specula, then stored in freezers at −80 °C until the tests were performed. After collecting the material from all patients participating in the study, the material was transferred into thermoboxes intended for transporting biological material for testing. After thawing, the concentration of interleukin-6 in vaginal secretions was determined using the Atellica IM IL6 test (Siemens Healthcare GmbH, 91052 Erlangen, Germany). This is a fully automated, single-stage, direct immunological test using the chemiluminescence technique. During the examination, a swab from the cervical canal was also taken in the speculums to be cultured for aerobic bacteria and fungi, anaerobic and atypical pathogens: *Ureaplasma* spp., *Mycoplasma hominis*, *Chlamydia trachomatis*. Solid media were used to culture the cervical canal secretion for aerobic and anaerobic bacteria and fungi. The test for atypical pathogens was performed using the MYCOPLASMA IST 3 test by Biomerieux (Biomerieux SA, 69280 Marcy-l’Etoile-France), and for *Chlamydia trachomatis*, the NADAL Chlamydia Test cassette (nal von minden GmbH, 47445 Moers, Germany) was used. A cervical swab for the Real-Time PCR test for *Ureaplasma urealyticum*, *Mycoplasma hominis* and *Chlamydia trachomatis* was collected using a dedicated swab and then stored in a freezer at −25 °C. During the cesarean section, 25 mL of amniotic fluid was collected, from which 8 mL was poured onto liquid media for the culture of aerobic bacteria and fungi and anaerobic bacteria, and 5 mL of fluid was frozen at −25 °C for testing for atypical pathogens using the Real-Time PCR method. This material, like the cervical swabs, was sent to the laboratory in transport boxes. The remaining amniotic fluid was frozen in test tubes and stored at −80 °C until the following tests were performed: glucose, Il-6 and PCT. After collecting the material from all patients participating in the study, the material was transferred into thermoboxes intended for transporting biological material. The concentration of Il-6 in the amniotic fluid was determined using the Atellica IM IL6 test, the concentration of PCT in the amniotic fluid was determined using the Atellica IM BRAHMS PCT test (ATELLICA® IM B·R·A·H·M·S PCT™ assay), and the glucose concentration was determined using the Atellica CH Glucose Hexokinase_3 test.

Then, after the afterbirth was extracted during cesarean section, a fragment of the placenta and fetal membranes was collected and stored in freezers at −80 °C until the histopathological examination was performed. After collecting the material from all patients participating in the study, the placental fragments were thawed and fixed in 10% formalin. The cut sections were processed in a Leica ASP300 S tissue processor (Leica Biosystems Nussloch GmbH) and then embedded in paraffin. Sections were prepared from the prepared paraffin blocks and placed on glass slides. In the next stage, the slides were placed in a 70 °C incubator for 2 h. After deparaffinizing the sections, they were stained with hematoxylin and eosin. The material was assessed by a pathologist using an Olympus BX46 light microscope (Olympus, Hamburg, Germany) for the presence of inflammatory infiltration within the fetal membranes and placenta. Based on the results, the patients were divided into two groups: the study group with IAI and the control group without IAI.

EONI was diagnosed by neonatologists up to 72 h of life based on a comprehensive clinical assessment of the child (signs of respiratory distress, the need for mechanical ventilation, irritability, or lethargy, and feeding intolerance, cyanosis, brady/tachycardia, acidosis), taking into account perinatal risk factors for congenital infection, and the results of additional tests routinely used in clinical practice at this hospital: WBC, PCT, Il-6, CRP, and blood culture. Due to the influence of prenatal antibiotic therapy, a positive blood culture from the child was not necessary to diagnose EONI. In newborns from the control group, tests were performed only in cord blood. In newborns without confirmed EONI, antibiotic therapy was discontinued after 48–72 h [12].

Statistical analysis: Statistical analysis was performed using the “R 4.0.2” program and the statistical program STATA 11 license number 30110532736. The Shapiro–Wilk test was used to check the normality of the distribution. In the vast majority of cases, the distribution deviated from the norm; therefore, nonparametric tests were used in the calculations. The results were presented as medians and interquartile ranges (IQR). The Mann–Whitney U test was used to compare differences between groups. The risk of intrauterine infection was assessed using univariate logistic regressions, and the results were presented as odds ratio (OR) and 95% confidence interval (95% CI). The analysis of the results of microbiological cultures and the result of the histopathological examination was estimated using the McNemmar test. *p* values < 0.05 in all analyses were considered statistically significant.

## 3. Results

All patients from the study group were in a preterm pregnancy on the day of delivery. In the control group, 27.7% were patients with a pregnancy below 37 weeks. Table 1.

The group B Streptococcus (GBS) carrier status of the patients from the study group was unknown due to their gestational age. According to the recommendations on pregnancy management, a vaginal and rectal swab should be taken between 36 0/7 and 37 6/7 weeks’ gestation [13]. In the case of the control group, a significant number of them had indications for a planned cesarean section, did not have the result on admission to the hospital or did not have a test for *Streptococcus agalactiae*; therefore, the GBS carrier status in the groups of patients included in the study is unknown.

All patients participating in the study were delivered by cesarean section. In the study group, 55.9% (19/34) of them had indications of suspected intrauterine infection. The second group of indications was preterm labor in progress in 41.2% (14/34) of women with an additional indication for termination of pregnancy, i.e., risk of intrauterine fetal hypoxia or other than cephalic fetal position, gestational diabetes, or hypertension. In one case, the decision to terminate the pregnancy prematurely by cesarean section was based on psychiatric indications. In the control group, the most common indication for termination of pregnancy by cesarean section was tokophobia (in 30.5% of patients, 11/36), and the remaining ones included no progress in labor (25%, 9/36), risk of intrauterine fetal hypoxia (8%), condition after cesarean section (19%), preeclampsia (4%), diabetes (5,5%), and others: ophthalmological, orthopedic maternal indications (8%).

In both groups (HY and HN), prenatal antibiotic therapy was used in 28/70 (40%) cases due to the risk of intrauterine infection and preterm delivery. Ampicillin or erythromycin and clindamycin were used 2–4 days before delivery. All patients received antibiotic prophylaxis before cesarean section with cefazolin.

In 91.5% (31/34) of newborns from the group of patients with histologically confirmed intra-amniotic infection (HY), early-onset neonatal infection was diagnosed based on the analysis of the documentation kept by the team of neonatologists. In three cases, clinical symptoms in newborns (respiratory disorders, food intolerance) were due to prematurity; infection was not confirmed. All infants with EONI received a full course of antibiotics for 7–14 days and recovered well. No newborn died.

### 3.1. Analysis of Inflammatory Parameters in Blood, Vaginal Secretions and Amniotic Fluid

Analysis of inflammatory parameters in selected body fluids showed that the most significant differences between groups can be observed in the case of IL-6 concentration in blood, vaginal secretions (VS) and amniotic fluid (AF). The results of differences in all inflammatory parameters are presented in Table 2.

The results of logistic regression are presented in Table 3.

To establish the cut-off values of continuous variables for chorioamnionitis and afterbirth inflammation in the histopathological examination, in patients without significant macroscopic changes (reference group HN), the analysis of ROC curves (receiver operating characteristic curves) was used. The results were described by providing the area under the curve (AUC), standard error of AUC (SD), 95% confidence interval for AUC (95% CI), probability *p*, and coordinates of ROC curves, i.e., for individual ranges of values of the continuous variable, the sensitivity of the ‘inflammation’ group and the specificity of the reference group were estimated. Table 4.

### 3.2. Assessment of the Usefulness of Microbiological Tests in Diagnosing Intrauterine Infection

In all patients, microbiological cultures of cervical smear and amniotic fluid were performed for aerobic bacteria, fungi and anaerobic bacteria.

Cervical swab cultures were sterile in 75.7% of all cases studied, in the study and control groups (53/70). In the study group, in 13/34 women, the cultures were positive: Candida albicans was grown in 4, GBS in 3, E. coli in 2, and in 4 women there was a mixed flora of fungal flora (Candida albicans) and aerobic bacteria (GBS, E. coli, Staphylococcus epidermidis). In the control group, 4 women had positive cervical smear cultures: Candida albicans and GBS twice each. In the case of amniotic fluid culture, the most common pathogens were aerobic bacteria (GBS—2; Escherichia coli—2), and this problem affected 5.7% of all patients.

One of the patients in the control group (HN) had a positive result of the aerobic amniotic fluid culture. Homogeneous growth of *Escherichia coli* was obtained, despite the lack of signs of inflammation in the histopathological examination of the afterbirth. Based on the analysis of the medical records of the newborn born to the mother with the above-mentioned result, no congenital infection was found. The newborn’s follow-up was also negative for infection. Perhaps the presence of these bacteria in the amniotic fluid occurred shortly before delivery, before the inflammation developed, and the antibiotic used before the cesarean section protected the mother and the child?

All patients underwent a culture (using the Mycoplasma IST 3 and NADAL tests) from the cervical smear for *Ureaplasma* spp., *Mycoplasma hominis* and *Chlamydia trachomatis*, as well as PCR analysis of the cervical smear and amniotic fluid for these atypical pathogens. The most common pathogen, regardless of the method and material tested, turned out to be atypical *Ureaplasma* spp. The presence of *Ureaplasma* spp. was detected in 26% of cervical smear preparations performed by culture and over 30% by PCR. The results of the statistical analysis showed that there are no significant differences between the culture method and the PCR method (*p* = 0.13). This means that these methods gave comparable results. In the case of amniotic fluid, the presence of *Ureaplasma urealyticum* was detected in less than 5% of preparations. No atypical pathogens such as *Mycoplasma hominis* and *Chlamydia trachomatis* were detected in the amniotic fluid.

Blood cultures of newborns were positive in only 5 of 31 infected cases: 2 cases each of GBS and *E. coli* and 1 case of GBS infection with *Ureaplasma urealitycum*.

### 3.3. Analysis of the Results of the Histopathological Examination

Histopathological examination of the afterbirths did not reveal any inflammatory changes in any of the evaluated placentas. Inflammatory infiltrates were found in 34 cases of examined fetal membranes and umbilical cords.

Based on the results of the histopathological examination, the patients were divided into those with intra-amniotic infection (study group HY) and those without infection (control group HN).

In the analyzed material, only 5 positive results of amniotic fluid cultures were obtained in the study HY group (5/34). Therefore, the difference between the diagnosis of intra-amniotic infection using the amniotic fluid culture method and the histopathological examination method was significant (*p* < 0.05). A significant number of negative culture results in the study group could be related to prenatal antibiotic therapy and skin flora contamination with prelaboratory errors.

## 4. Discussion

Intra-amniotic infection is the most common cause of preterm birth [13,14]. Routinely determined in clinical practice, concentrations of biochemical parameters (CRP, PCT, WBC) of inflammation turn out to be of low usefulness in IAI diagnostics [7]. The inflammatory process most often develops in the ascending route, encompassing the vagina, cervix, and contents of the uterine cavity, including the fetus, fetal membranes, umbilical cord, and placenta. The development of infection can take a long time locally, especially when antibiotics are used in the pregnant woman, or it can be rapid. In such cases, premature labor and the birth of an infected child most often occur. Valuable clues are provided by determining inflammatory parameters in vaginal secretions and amniotic fluid. In our study, we obtained positive blood cultures only in (5/31) neonates who were diagnosed as having been treated with EONI. This is consistent with reports from other authors [14,15]. Inflammatory changes of the afterbirth were found in 26% of cases.

### 4.1. Determination of Whether CRP Concentration in Blood Is Associated with the Occurrence of Intrauterine Infection

In our own studies, the median CRP blood concentration of patients in the study group was 7.36 mg/L, while in the control group it was 2.99 mg/L, and this difference was not significant (*p* = 0.15). The results obtained using logistic regression showed that an increase in CRP blood level does not increase the risk of IAI (OR-1.23; 95% CI: 1.24–1.89; *p* = 0.34). ROC curve analysis showed an AUC of 0.63 (*p* = 0.2), sensitivity of 58%, and specificity of 89%.

Different results were obtained by Caloone et al., who stated that blood CRP is a predictor of chorioamnionitis in histopathological examination in patients with premature rupture of membranes between 22–36 + 6 weeks of gestation. In their study in patients with inflammatory changes, the CRP blood level was significantly higher than in patients without inflammation (10.1 vs. 4 mg/mL). ROC curve analysis showed AUC = 0.7 [15]. Similar results were presented by Yoon et al., who showed that the median CRP blood concentration was significantly higher in patients with than without histopathological inflammation (0.4 vs. 1.2 mg/dL; AUC = 0.79). A concentration of 0.7 mg/dL was associated with a sensitivity of 0.81 in identifying acute postpartum inflammation [16].

Torbe et al. attempted to determine the usefulness of CRP determination in the blood serum of mothers with pPROM in predicting the occurrence of congenital infection in the newborn. The mean serum CRP concentration of mothers giving birth to a child with congenital infection was 9.4 mg/L compared to 6.3 mg/L to those who delivered healthy newborns. Two cut-off points, ≥10 mg/L and ≥15 mg/L, showed sensitivity, specificity, PPV and NPV in predicting congenital infection of 47% vs. 47%, 63% vs. 76%, 38% vs. 47%, and 72% vs. 76%, respectively. In conclusion, the authors stated that mothers’ serum CRP levels have low diagnostic value in predicting the occurrence of congenital infection in newborns [17]. Similar results were obtained by Martius et al. The results of logistic regression analysis showed that the concentration of CRP > 15 mg/L does not increase the risk of congenital infection in the newborn [18].

### 4.2. Determination of Whether the Concentration of IL-6 in Blood, Vaginal Secretions and Amniotic Fluid Is Associated with the Occurrence of Intrauterine Infection

In our study, the median concentration of serum interleukin-6 was significantly higher in the study group than in the control group (6.2 pg/mL vs. 2.6 pg/mL; *p* = 0.008). The logistic regression analysis showed that rising IL-6 blood level increases the risk of IAI (OR = 1.25, 95% CI: 1.062–1.52, *p* = 0.012). ROC analysis showed AUC = 0.72 (*p* = 0.03), a cut-off point of 4.55 pg/mL, sensitivity 75% and specificity 85%.

Park et al. evaluated the value of selected inflammatory cytokine determination, including Il-6, in maternal blood and amniotic fluid in prediction of IAI and spontaneous preterm labor in patients at gestational age 23–33 + 6 weeks with diagnosed preterm labor. Intrauterine infection was diagnosed based on amniotic fluid culture. Il-6 concentration in patients with positive fluid culture was significantly higher (9.9 vs. 4.6 pg/mL). In addition, logistic regression analysis showed that high levels of IL-6 (>4.8 pg/mL) in maternal plasma were significantly associated with impending preterm delivery [19]. The study of Cobo et al. performed in women with preterm delivery and in women with pPROM, both at 22–33 + 6 weeks of gestation, provides slightly different information. In patients with premature rupture of the membrane and microbes in the amniotic fluid, the median for IL-6 was 19 pg/mL, while in the absence of pathogens in the fluid, it was 16 pg/mL, but the difference was not significant. In the group of patients with premature labor and positive amniotic fluid, the median for IL-6 was 18 pg/mL, and in patients with negative microbiological test results, it was 16 pg/mL and the differences were also not significant. Only after dividing the patients into groups according to gestational age were statistically significant results obtained; namely, in the case of patients with preterm delivery at gestational age 22–31 + 6 weeks of gestation, the median for IL-6 was 21 pg/mL in women with infection in the amniotic fluid and 14 pg/mL in patients without infection [20]. In turn, Kopyra et al. in a study assessing the usefulness of Il-6 in predicting IAI and newborn condition in pregnant women with PROM showed that in children of mothers with Il-6 levels exceeding 5 pg/mL, symptoms of congenital infection were significantly more frequent than in patients with Il-6 levels below 5 pg/mL [21]. Martinez-Portilla et al. conducted a study in patients between 26–36 + 6 weeks of gestation with pPROM to evaluate the value of the assessment of Il-6 concentration in maternal blood in predicting histopathological chorioamnionitis. In 66% of included patients, histological chorioamnionitis was diagnosed and the Il-6 level of 19.5 pg/mL was the best cut-off point for predicting chorioamnionitis [22].

Interleukin-6 also plays a significant role in predicting the occurrence of impending preterm labor. Mutrha et al. studied the Il-6 level in patients with preterm pregnancies with an increased risk of preterm labor (at least 4 contractions/hour or pPROM) and without risk factors for preterm labor. It was shown that in patients without the risk of preterm labor, the Il-6 value was below 8 pg/mL, while in patients with risk factors, it was ≥8 pg/mL, and in such cases the median time interval from the moment of sample collection to delivery was significantly shorter [23]. Kopyra et al. in the previously cited work obtained the highest predictive value for Il-6 in relation to the duration of pregnancy from the moment of sample collection to delivery. In the case of 7 days the AUC ROC value was 0.69, for 3 days 0.582, and for 24 h 0.649. It was established that the Il-6 concentration of 7 pg/mL is the cut-off point for dividing patients into low- and high-risk groups of delivery within 7 days. Based on reports, it can be stated that the level of Il-6 seems to be elevated in patients with expected preterm labor. This may prove useful in the treatment of patients with risk factors for preterm labor [21].

Our studies showed significantly higher values of IL-6 in vaginal fluid (9.65 pg/mL) in the study group than in the controls (2.7 pg/mL, *p* < 0.0001). The logistic regression analysis showed that a higher Il-6 value in the vaginal fluid increases the risk of IAI (OR = 1.59, 95% CI: 1.23–2.32, *p* = 0.003). The ROC curve showed AUC 0.95; *p* < 00001; cut-off point 6.05 mg/dL; sensitivity 92%; specificity 96%.

El Ghazaly et al. stated that the bedside test, which assessed IL-6 in vaginal secretion, is accurate and non-invasive with 98.6% sensitivity, 94.7% specificity, 97.3% PPV, 97.3% NPV, and 97.3% overall accuracy in detecting chorioamnionitis. The IL-6 bedside test had significantly higher sensitivity, specificity, NPV, and overall accuracy than the clinical and laboratory parameters of chorioamnionitis [24].

In our own studies, significantly higher values of Il-6 in amniotic fluid were found (920 pg/mL) in the study group compared to the control group (221 pg/mL, *p* = 0.04). Logistic regression analysis showed that in the case of increased Il-6 in amniotic fluid, the risk of IAI is higher.

Romero et al. tried to determine the diagnostic and prognostic value of selected markers, including Il-6 in amniotic fluid in patients with preterm labor and with intact fetal membranes. The median Il-6 in amniotic fluid was significantly higher in patients with IAI than in those without (35.9 ng/mL vs. 1.7 ng/mL). In their study 9.2% of patients had a positive result of amniotic fluid culture. The best cut-off point for IL-6 was 11.3 ng/mL. Additionally, it was found that the time from amniocentesis to delivery was shorter in patients with positive amniotic fluid culture compared to patients with negative culture. The cited research confirms the diagnostic value of Il-6 in detecting subclinical invasion of the amniotic cavity and the prognostic value in predicting the occurrence of preterm delivery [25].

### 4.3. Determining Whether Procalcitonin Concentration in Blood and Amniotic Fluid Is Associated with the Occurrence of Intrauterine Infection

Diagnosis of IAI based only on clinical symptoms is characterized by low sensitivity and specificity. Therefore, many studies are being conducted to find an appropriate marker of intrauterine infection in asymptomatic patients. In our own studies, the level of procalcitonin in the peripheral blood of patients with IAI did not differ from the level in patients without infection (0.06 ng/mL vs. 0.05 ng/mL, *p* = 0.19). ROC analysis showed AUC 0.66, *p* = 0.12, cut-off point 0.055 ng/mL, sensitivity 67%, and specificity 70%.

Oludag et al. conducted studies on the use of PCT concentration in the peripheral blood of patients with premature rupture of the amniotic sac in predicting subclinical IAI. The mean PCT level in patients between 24–34 weeks of gestation with pPROM was significantly higher (0.086 ng/mL) than in patients in the control group (0.033 ng/mL). Additionally, it was found that in the study group, the procalcitonin level in patients with retrospectively diagnosed chorioamnionitis in the histopathological examination was significantly higher compared to patients without inflammatory infiltration in the fetal membranes. The cut-off value for PCT in predicting histological chorioamnionitis showed that the highest sensitivity and specificity corresponded to a concentration of 0.054 ng/mL. Sensitivity in predicting chorioamnionitis in the histopathological examination was 92.3%, specificity 68.4%, positive predictive value 66.7%, and negative predictive value 92.9%. In addition, it was found that the mean PCT values in patients who gave birth to newborns with congenital infection did not differ significantly compared to patients who gave birth to newborns without infection [26]. Kopyra et al. assessed the usefulness of inflammatory parameters, including PCT, in predicting IAI and the condition of the newborn in patients with ruptured amniotic sacs. Procalcitonin had the highest predictive value in predicting the occurrence of congenital infection in a newborn. The best cut-off point for PCT was 0.5 ng/mL, which allowed for dividing patients into groups of patients with high and low risk of giving birth to an infected newborn [21].

In turn, Torbe compared blood PCT values in patients with preterm pregnancies with pPROM and PROM at term with healthy patients with preterm and full-term pregnancies in order to detect IAI. While PCT values in patients with pPROM and with PROM at term were significantly increased compared to the levels in healthy patients, these values in patients with pPROM and PROM at term were comparable. According to the authors, the PCT limit value of 1.9 ng/mL allows for the prediction of congenital infection in the newborn with a sensitivity of 53% and for the prediction of chorioamnionitis in histopathological examination with a sensitivity of 75%. Specificity, however, was 45% and 55%, respectively [27]. Considering the conflicting reports on the usefulness of PCT concentration determination in peripheral blood, it should be stated that it requires conducting studies on a larger group of patients.

### 4.4. Determination of Whether Glucose Concentration in Amniotic Fluid Is Associated with the Occurrence of Intraamniotic Infection

Statistical analysis of our own studies showed that the glucose level in amniotic fluid is associated with the occurrence of IAI. The median for glucose in amniotic fluid was 16 mg/dL in the study group and 25 mg/dL in the control group (*p* < 0.002). The results of logistic regression analysis showed that an increase in glucose value in amniotic fluid reduces the risk of IAI (OR = 0.749, 95% CI: 0.6–0.86, *p* = 0.001). The ROC curve showed AUC 0.93, *p* < 00001, cut-off point 18.5 mg/dL, sensitivity 100%, and specificity 80%.

The role of glucose as a marker of ongoing infection in amniotic fluid has been widely described. Kacerovsky et al. conducted a study evaluating glucose levels in amniotic fluid in women with premature rupture of the membranes in preterm pregnancies. They divided the patients into groups depending on the presence of microorganisms in the amniotic cavity and/or intraamniotic inflammation. In patients with intrauterine infection (presence of pathogens in amniotic fluid and intraamniotic inflammation), the glucose level was significantly lower (median 11.6 mg/dL). In the case of sterile intraamniotic inflammation (presence of Il-6 > 3000 pg/mL without detected microbial invasion in the amniotic fluid), the median glucose concentration was 6.3 mg/dL. In the case of amniotic fluid colonization (a group of patients in whom, despite the detection of pathogens in the amniotic fluid, no elevated Il-6 level was found), as well as in women with negative amniotic fluid (no microorganisms in the amniotic fluid and no inflammation), the median glucose level was 21.6 mg/dL vs. 23.4 mg/dL. A significant difference was found in the glucose level between patients with IAI, intraamniotic inflammation, amniotic fluid colonization and negative amniotic fluid. In the cited study, patients with IAI and those with intra-amniotic inflammation had lower glucose levels than patients with colonization and negative amniotic fluid. A glucose concentration of 10 mg/dL was optimal for identifying intra-amniotic inflammation in patients with pPROM. Additionally, it was shown that glucose levels in amniotic fluid do not differ significantly depending on the type of pathogen found in the amniotic fluid [28]. Odibo et al. looked for an association between glucose concentration in amniotic fluid and histopathological chorioamnionitis in patients with preterm labor and intact membranes between 22 and 35 weeks of gestation. In this study, positive amniotic fluid cultures were obtained in 17% of cases, while histological chorioamnionitis was found in 60.2% of cases. It was determined that glucose level < 20 mg/dL is the most sensitive predictor of histological chorioamnionitis [29]. The collected data clearly show that invasion of pathogens into the amniotic cavity results in a decrease in glucose level in amniotic fluid. This is most likely related to glucose metabolism by white blood cells and bacteria.

### 4.5. Assessment of the Usefulness of Microbiological Tests in the Diagnosis of Intrauterine Infection

In our own studies, among the patients from the study group, as many as 42% had a positive culture from the cervical canal for *Ureaplasma* spp., and the PCR method confirmed the presence of this pathogen in 50%. In the control group, it was 21% for cultures and 23% for the PCR method, respectively. The results regarding *Mycoplasma hominis* were as follows: in the study group, a positive culture was found in 17% of cases, and in the control group, a positive culture was found in 3%. In the case of the PCR method, all patients from the study group had a negative result, while 3% of patients from the control group had a positive result. Some discrepancies between the number of positive cultures for atypical bacteria and the number of positive PCR results are due to the methodological requirements of the study. Obtaining bacterial growth on a culture medium requires the presence of live microorganisms in the material, whereas for PCR testing, a bacterial fragment is sufficient. Zbrodowska-Stefanow et al. assessed the frequency of *Ureaplasma urealyticum* and *Mycoplasma hominis* infections in non-pregnant women using the culture method. Their results show that *Ureaplasma urealyticum* occurs in 29.8% of the studied women, while *Mycoplasma hominis* occurs in 3.7% of the studied cases [30]. Data from the literature show that the occurrence of urogenital mycoplasmas depends on, among others, age, sexual activity and pregnancy. Kacerovsky et al. conducted a study in patients between 22–34 + 6 weeks of gestation, with premature delivery and preserved fetal membranes. In this study, the presence of *Ureaplasma* spp. determined by the PCR method was demonstrated in 51% of the patients included in the study. The presence of *Ureaplasma* spp. in the cervix was found much more often in patients with diagnosed IAI than without (75% vs. 36%). The simultaneous occurrence of *Ureaplasma* spp. and *Mycoplasma hominis* in the cervix was found more often in patients with IAI than without (42% vs. 7%) [31].

Miyoshi et al. demonstrated that a positive vaginal examination for *U. urealyticum*/*M. hominis* in patients with a risk of preterm labor was an independent predictor of delivery before 37 weeks of gestation. This suggests the value of routine vaginal examination for *U. urealyticum*/*M. hominis* in patients at risk of preterm labor [32].

In the present study, attention is drawn to the negligible number of positive amniotic fluid cultures in the study group.

In our study, prenatal antibiotic therapy was not relevant for the results of cultures of aerobic and anaerobic bacteria and atypical bacteria in any of the biological materials (VF or AF). Also, the results of prenatal antibiotic therapy in conjunction with PCR were not significant.

### 4.6. Analysis of the Usefulness of Histopathological Examinations of the Afterbirth in Diagnosing Intrauterine Infection

In our study, 26% of patients showed inflammatory changes in the histopathological examination of the afterbirth. Based on the presence of inflammatory infiltration in the histopathological examination, the patients were divided into groups: the study group of patients with IAI and the control group of patients without IAI. Due to the use of antibiotics during cesarean section, microbiological methods seemed imperfect; therefore, it was decided to divide the patients into groups in this way.

Caloone et al. conducted a study among patients diagnosed with pPROM between 22–36 + 6 weeks of pregnancy to assess selected maternal markers in predicting the occurrence of IAI diagnosed on the basis of histopathological examination of the afterbirth. The author showed that out of 295 patients participating in the study, 42.7% were diagnosed with IAI based on the histopathological examination. Children born to patients with IAI are statistically smaller than those born to patients without chorioamnionitis [16]. Wu et al. showed that chorioamnionitis in histopathological examination is associated with the occurrence of preterm delivery and with increased neonatal morbidity and mortality. The results of their analyses also clearly show that histopathological chorioamnionitis is associated with a higher concentration of C-reactive protein in the newborn and a lower gestational age [33]. Lu et al. showed that severe chorioamnionitis and increased levels of proinflammatory cytokines (IL-6, IL-8, TNF-alpha, G-CSF in cord blood) were associated with an increased risk of brain damage in premature infants [34].

Katz et al. conducted a study assessing the afterbirths of patients with premature rupture of the amniotic sac and analyzing the relationship between the result of the histopathological examination and CRP, gestational age, birth weight of the newborn and the Apgar score. In 50% of the afterbirths examined, features of histopathological chorioamnionitis were found. In premature infants born to mothers with chorioamnionitis, in histopathological examination, gestational age, birth weight and Apgar score were significantly lower than in infants born to mothers without signs of inflammation in postnatal examination. On the other hand, CRP concentration of newborns measured immediately after delivery and 12 h later was significantly higher in infants born to mothers with inflammation in histopathological examination [35].

### 4.7. Analysis of the Relationship Between the Diagnosis of Intrauterine Infection and the Occurrence of Congenital Infection in the Newborn

In our own studies, among the group with diagnosed IAI based on histopathological examination, congenital infection in the newborn was confirmed in 91.66% of cases, while in the control group, it was confirmed in 8.82%.

Budal et al. showed that in the case of post-term births the rate of histologic chorioamnionitis (HCA), fetal inflammatory response, and clinical chorioamnionitis was significantly higher, and post-term newborns were treated in the NICU more often than those born near term [36].

Ramsey et al. showed that the gestational age among patients with diagnosed chorioamnionitis was significantly lower compared to patients without this diagnosis. In 13% of patients with pPROM between 24 + 0 and 36 + 6 weeks of pregnancy, a diagnosis of chorioamnionitis was made. Serious morbidity was defined as neonatal sepsis, meningitis, respiratory distress syndrome, grade 3 and 4 intraventricular hemorrhage, or pneumonia, among others. Children of patients with chorioamnionitis compared to those without chorioamnionitis were characterized by lower birth weight, lower Apgar score at 1 min, and more frequent occurrence of serious neonatal complications mentioned above [37]. Rallis et al. show that newborns with confirmed intra-amniotic infection (HCA) and advanced FIRS are at increased risk of early-onset sepsis after taking into account other perinatal and neonatal factors [38].

Oshima et al. conducted a study in patients over 22 weeks of gestation with suspected IAI. Congenital infection was diagnosed in 9/200 neonates. In all mothers whose children were diagnosed with congenital infection, IAI was confirmed by histopathological examination of the afterbirth. Among these patients, the median amniotic fluid Il-6 concentration was significantly higher compared to patients giving birth to children without congenital infection [39].

The difference in the number of intra-amniotic infections and diagnosed EONI in newborns may indicate the presence of mechanisms limiting infection in the newborn or the presence of mechanisms limiting infection in the amniotic cavity. It is also possible that prenatal antibiotic therapy had an inhibiting effect on the development of infection in the child. There is also a risk of failure to recognize the infection within 72 h of the child’s birth, although in the case of premature babies staying in hospital longer, this is less likely. On the other hand, in the group of newborns of mothers from the control group (HN), EONI was diagnosed in 8.3% (3/36) of cases, despite the lack of diagnosis of IAI in the mother. This may have concerned the very early phase of infection, which in the patient had not yet caused changes in the afterbirth, while in the newborn, congenital infection developed.

A strength of our work is the wide selection of biological materials examined and the use of biochemical, microbiological, molecular, and histopathological methods. We recognize that the limited number of cases analyzed requires confirmation of the results in a larger population. The mechanisms determining the development of intrauterine infection and early-onset infection in the newborn are extremely complex and still require research.

## 5. Conclusions

Our studies support that mothers’ serum CRP assessments as well as serum PCT assessments are of poor diagnostic value in the prediction of IAI confirmed in histopathological examination. The best predictive value of IAI confirmed in histopathological examination was vaginal fluid IL-6 determinations.

## Figures and Tables

**Table 1:** Maternal and neonatal characteristics of the study population.

	Study Group HY; *n* = 34	Control Group HN; *n* = 36	*p*
**Mother’s parameters**			
Age [years]; X ± SD	29.3 ± 4.8	32.6 ± 4.9	**0.02**
Previous pregnancies [number]	2.6 ± 1.8	2.2 ± 1.1	NS
Previous births [number]	2.1 ± 1.2	1.8 ± 0.8	NS
Body mass [kg]	69.6 ± 10.6	83.0 ± 15.6	**0.002**
Height [cm]	166.2 ± 4.2.0	167.3 ± 6.0	NS
BMI [kg/m^2^]	25.2 ± 3.9	29.7 ± 5.2	**0.005**
Patient’s body temperature [°C]	36.7 ± 0.4	36.4 ± 0.3	NS
Antepartum maternal antibiotics N [%]	25 (3.5)	3 (8.3)	**<0.05**
pPROM; N [%]	16 (47.1)	3 (8.3)	**<0.05**
PIH; N [%]	3 (8.8)	4 (11.1)	NS
GDM; N [%]	6 (17.6)	16 (44.4)	NS
Prenatal steroid therapy; N [%]	31 (91.5)	8 (22.2)	**<0.05**
**Newborn’s parameters**			
Gestational age [weeks]	30.1 ± 2	37.6 ± 3.1	**<0.0001**
Birth weight [g]	1538.6 ± 453.3	3371.2 ± 492.3	**<0.0001**
Birth length [cm]	42.3 ± 4.3	53.3 ± 2.5	**<0.0001**
Hospital stay [days]	40 ± 21.1	2.5 ± 1.6	**<0.0001**
Apgar score in 5 min [points]	7.5 ± 0.8	9.3 ± 0.8	**0.00003**
Early onset neonatal infection; N [%]	31 (91.5)	3 (8.3)	**<0.005**
Blood culture positive; N [%]	5 (14.7)	0	**<0.001**
Clinical symptoms; N [%]	33 (97)	3 (8.3)	**<0.001**
CRP at birth [mg/L] X ± SD	11 ± 10	3 ± 2.5	**<0.05**
WBC at birth [cells/mm^3^]	11.72 ± 8.33	12.67 ± 7.52	NS
PCT at birth [ng/mL]	6.22 ± 1.243	0.125 ± 0.091	**<0.001**
Il-6 at birth [pg/mL]	2360 ± 1743	13 ± 6	**<0.0001**

Legend: X—mean; SD—standard deviation; BMI—body mass index; NS—not significant; pPROM—premature rupture of amniotic membranes before 37 weeks of gestation; PIH—pregnancy-induced hypertension; GDM—gestational diabetes mellitus; CRP—C-reactive protein; PCT—procalcitonin; Il-6—interleukin 6.

**Table 2:** Concentration values of inflammatory parameters in individual body fluids.

Type of Material	Tested Parameter	Study Group HY *n* = 34		Control Group HN *n* = 36		*p*
		Me	IQR	Me	IQR	
Venous blood	Il-6 (pg/mL)	6.3	9.2	2.6	1.4	**0.008**
	PCT (ng/mL)	0.06	0.07	0.05	0.02	0.19
	CRP (mg/L)	7.36	17.08	2.99	3.19	0.15
Amniotic fluid (AF)	Il-6 (pg/mL)	920	5321.9	221.8	91.6	**0.04**
	PCT (ng/mL)	0.07	0.05	0.06	0.03	**0.002**
	Glucose (mg/dL)	16	11	25	11	**0.002**
Vaginal fluid (VF)	Il-6 (pg/mL)	9.65	20.95	2.7	0	***p*** **< 0.0001**

Legend: Me—median; IQR—interquartile range; CRP—C-reactive protein; PCT—procalcitonin; Il-6—interleukin-6.

**Table 3:** Results of logistic regression analysis of the risk of intrauterine infection.

Logistic Regression Analysis of the Risk of Intrauterine Infection			
Parameter	OR	95% CI	*p*
CRP in blood (mg/l)	1.23	1.24–1.89	0.34
Il-6 in blood (pg/mL)	1.25	1.062–1.52	**0.012**
Il-6 in vaginal discharge (pg/mL)	1.59	1.23–2.32	**0.003**
Il-6 in amniotic fluid (pg/mL)	1.01	1.0004–1.06	**0.01**
PCT in blood (ng/mL)	1.3	1.02–1.17	0.6
PCT in amniotic fluid (ng/mL)	3.72	1.67–4.59	**0.001**
Glucose in amniotic fluid (mg/dL)	0.75	0.6–0.86	**0.001**

Legend: CRP—C-reactive protein; Il-6—interleukin 6; PCT—procalcitonin.

**Table 4:** ROC analysis for the tested parameters: AUC, cut-off point, SE, SP.

Parameter	AUC	SD	95% Cl	*p*	Cut Off Point	SE	SP
Blood CRP	0.63	0.12	0.56–0.93	0.2044	7.82	58	89
Blood Il-6	0.72	0.12	0.49–0.94	**0.0319**	4.55	75	85
Il-6 VF	0.95	0.05	0.85–1.05	**<0.00001**	6.05	92	96
Il-6 AF	0.88	0.08	0.72–1.04	**0.0001**	1100	83	97
Blood PCT	0.66	0.1	0.47–0.85	0.12	0.055	67	70
PCT AF	0.79	0.09	0.62–0.96	**0.0041**	0.085	67	87
Glucose AF	0.93	0.04	0.86–1.01	**<0.00001**	18.5	100	80

Legend: Il-6—interleukin 6 concentration (pg/mL); PCT—procalcitonin concentration (ng/mL); CRP—C-reactive protein concentration (mg/L); AUC—area under curve; SD—standard deviation; CI—confidence interval; SE—sensitivity (%); SP—specificity (%); VF—vaginal fluid; AF—amniotic fluid.

## Data Availability

The datasets used and analyzed during the current study are available from the corresponding author on reasonable request.

## Abbreviations

AF	amniotic fluid
AUC	area under the (ROC) curve
CI	confidence interval
CRP	C-reactive protein
EONI	early-onset neonatal infection
FIRS	Fetal Inflammatory Response Syndrome
HCA	histologic chorioamnionitis
IAI	intra-amniotic infection
Il-6	interleukin-6
PCT	procalcitonin
pPROM	preterm premature rupture of the membranes
ROC	receiver operating characteristic curve
SE	sensitivity
SP	specificity
WBC	white blood cell
VF	vaginal fluid

## References

[1] Harrison M.S., Goldenberg R.L. (2016). Global burden of prematurity. Semin. Fetal Neonatal Med..

[2] Rees P., Callan C., Chadda K.R., Vaal M., Diviney J., Sabti S., Harnden F., Gardiner J., Battersby C., Gale C. (2022). Preterm Brain Injury and Neurodevelopmental Outcomes: A Meta-analysis. Pediatrics.

[3] Honnorat M., Plaisant F., Serret-Larmande A., Claris O., Butin M. (2023). Neurodevelopmental Outcome at Two Years for Preterm Infants with Intraventricular Hemorrhage: A Case-Control Study. Pediatr. Neurol..

[4] Pascal A., de Bruyn N., Naulaers G., Ortibus E., Hanssen B., Oostra A., de Coen K., Sonnaert M., Cloet E., Casaer A. (2023). The Impact of Intraventricular Hemorrhage and Periventricular Leukomalacia on Mortality and Neurodevelopmental Outcome in Very Preterm and Very Low Birthweight Infants: A Prospective Population-based Cohort Study. J. Pediatr..

[5] Perry A.K., Rossi R.M., DeFranco E.A. (2019). Severe adverse maternal outcomes associated with chorioamnionitis. Am. J. Obstet. Gynecol. MFM.

[6] Gibbs R.S., Romero R., Hillier S.L., Eschenbach D.A., Sweet R.L. (1992). A review of premature birth and subclinical infection. Am. J. Obstet. Gynecol..

[7] Higgins R.D., Saade G., Polin R.A., Grobman W.A., Buhimschi I.A., Watterberg K., Silver R.M., Raju T.N.K. (2016). Chorioamnionitis Workshop Participants. Evaluation and Management of Women and Newborns with a Maternal Diagnosis of Chorioamnionitis: Summary of a Workshop. Obstet. Gynecol..

[8] Peng C.C., Chang J.H., Lin H.Y., Cheng P.J., Su B.H. (2018). Intrauterine inflammation, infection, or both (Triple I): A new concept for chorioamnionitis. Pediatr Neonatol..

[9] Grandi C., Salomão K.B., de Freitas S.F., Rocha P.R.H., Cavalli R.C., Cardoso V.C. (2024). Mid-pregnancy circulating cytokine levels, placental efficiency and their relationship with preterm birth. Rev. Bras. Ginecol. Obstet..

[10] Olguín-Ortega A., Figueroa-Damian R., Palafox-Vargas M.L., Reyes-Muñoz E. (2024). Risk of adverse perinatal outcomes among women with clinical and subclinical histopathological chorioamnionitis. Front. Med..

[11] Rezagholi P., Rasouli M.A., Zare S. (2024). Indications of Amniocentesis and its Early and Late Complications. Adv. Biomed. Res..

[12] Celik I.H., Hanna M., Canpolat F.E., Pammi M. (2022). Diagnosis of neonatal sepsis: The past, present and future. Pediatr. Res..

[13] Coggins S.A., Puopolo K.M. (2024). Neonatal Group B Streptococcus Disease. Pediatr Rev..

[14] Gomez-Lopez N., Galaz J., Miller D., Farias-Jofre M., Liu Z., Arenas-Hernandez M., Garcia-Flores V., Shaffer Z., Greenberg J.M., Theis K.R. (2022). The immunobiology of preterm labor and birth: Intra-amniotic inflammation or breakdown of maternal-fetal homeostasis. Reproduction.

[15] Jung E., Romero R., Suksai M., Gotsch F., Chaemsaithong P., Erez O., Conde-Agudelo A., Gomez-Lopez N., Berry S.M., Meyyazhagan A. (2024). Clinical chorioamnionitis at term: Definition, pathogenesis, microbiology, diagnosis, and treatment. Am. J. Obstet. Gynecol..

[16] Caloone J., Rabilloud M., Boutitie F., Traverse-Glehen A., Allias-Montmayeur F., Denis L., Boisson-Gaudin C., Hot I.J., Guerre P., Cortet M. (2016). Accuracy of Several Maternal Seric Markers for Predicting Histological Chorioamnionitis After Preterm Premature Rupture of Membranes: A Prospective and Multicentric Study. Eur. J. Obstet. Gynecol. Reprod. Biol..

[17] Torbé A., Kowalski K. (2010). Maternal Serum and Vaginal Fluid C-Reactive Protein Levels Do Not Predict Early-Onset Neonatal Infection in Preterm Premature Rupture of Membranes. J. Perinatol..

[18] Martius J.A., Roos T., Gora B., Oehler M.K., Schrod L., Papadopoulos T., Gross U. (1999). Risk Factors Associated with Early-Onset Sepsis in Premature Infants. Eur. J. Obstet. Gynecol. Reprod. Biol..

[19] Park H., Park K.H., Kim Y.M., Kook S.Y., Jeon S.J., Yoo H.N. (2018). Plasma Inflammatory and Immune Proteins as Predictors of Intra-Amniotic Infection and Spontaneous Preterm Delivery in Women with Preterm Labor: A Retrospective Study. BMC Pregnancy Childbirth.

[20] Cobo T., Tsiartas P., Kacerovsky M., Holst R.M., Hougaard D.M., Skogstrand K., Wennerholm U.B., Hagberg H., Jacobsson B. (2013). Maternal Inflammatory Response to Microbial Invasion of the Amniotic Cavity: Analyses of Multiple Proteins in the Maternal Serum. Acta Obstet. Gynecol. Scand..

[21] Kopyra P., Seremak-Mrozikiewicz A., Drews K. (2010). Usefulness of PCT, IL-6, CRP Measurement in the Prediction of Intraamniotic Infection and Newborn Status in Pregnant Women with Premature Rupture of Membranes. Ginekol. Pol..

[22] Martinez-Portilla R.J., Hawkins-Villarreal A., Alvarez-Ponce P., Chinolla-Arellano Z.L., Moreno-Espinosa A.L., Sandoval-Mejia A.L., Moreno-Uribe N. (2019). Maternal Serum Interleukin-6: A Non-Invasive Predictor of Histological Chorioamnionitis in Women with Preterm-Prelabor Rupture of Membranes. Fetal Diagn. Ther..

[23] Murtha A.P., Greig P.C., Jimmerson C.E., Herbert W.N. (1998). Maternal Serum Interleukin-6 Concentration as a Marker for Impending Preterm Delivery. Obstet. Gynecol..

[24] El-Ghazaly T.E., Abdelazim I.A., Elshabrawy A. (2022). Interleukin-6 Bedside Test in Detecting Chorioamnionitis in Women with Preterm Premature Rupture of Fetal Membranes. Ginekol. Pol..

[25] Romero R., Yoon B.H., Mazor M., Gomez R., Diamond M.P., Kenney J.S., Ramirez M., Fidel P.L., Sorokin Y., Cotton D. (1993). The Diagnostic and Prognostic Value of Amniotic Fluid White Blood Cell Count, Glucose, Interleukin-6, and Gram Stain in Patients with Preterm Labor and Intact Membranes. Am. J. Obstet. Gynecol..

[26] Oludag T., Gode F., Caglayan E., Saatli B., Okyay R.E., Altunyurt S. (2014). Value of Maternal Procalcitonin Levels for Predicting Subclinical Intra-Amniotic Infection in Preterm Premature Rupture of Membranes: Procalcitonin Levels in PPROM. J. Obstet. Gynaecol. Res..

[27] Torbé A. (2007). Maternal Plasma Procalcitonin Concentrations in Pregnancy Complicated by Preterm Premature Rupture of Membranes. Mediat. Inflamm..

[28] Kacerovsky M., Holeckova M., Stepan M., Gregor M., Vescicik P., Lesko D., Burckova H., Pliskova L., Bolehovska R., Andrys C. (2022). Amniotic Fluid Glucose Level in PPROM Pregnancies: A Glance at the Old Friend. J. Matern. Fetal Neonatal Med..

[29] Odibo A.O., Rodis J.F., Sanders M.M., Borgida A.F., Wilson M., Egan J.F., Campbell W.A. (1999). Relationship of Amniotic Fluid Markers of Intra-Amniotic Infection with Histopathology in Cases of Preterm Labor with Intact Membranes. J. Perinatol..

[30] Zdrodowska-Stefanow B., Kłosowska W.M., Ostaszewska-Puchalska I., Bułhak-Kozioł V., Kotowicz B. (2006). Ureaplasma Urealyticum and Mycoplasma Hominis Infection in Women with Urogenital Diseases. Adv. Med. Sci..

[31] Kacerovsky M., Stranik J., Kukla R., Bolehovska R., Bostik P., Matulova J., Stepan M., Hladky J., Jacobsson B., Musilova I. (2022). Intra-Amniotic Infection and Sterile Intra-Amniotic Inflammation in Women with Preterm Labor with Intact Membranes Are Associated with a Higher Rate of Ureaplasma Species DNA Presence in the Cervical Fluid. J. Matern. Fetal Neonatal Med..

[32] Miyoshi Y., Suga S., Sugimi S., Kurata N., Yamashita H., Yasuhi I. (2022). Vaginal *Ureaplasma urealyticum* or *Mycoplasma hominis* and preterm delivery in women with threatened preterm labor. J. Matern. Fetal Neonatal Med..

[33] Wu H.-C., Shen C.M., Wu Y.Y., Yuh Y.S., Kua K.E. (2009). Subclinical Histologic Chorioamnionitis and Related Clinical and Laboratory Parameters in Preterm Deliveries. Pediatr. Neonatol..

[34] Lu H.Y., Zhang Q., Wang Q.X., Lu J.Y. (2016). Contribution of Histologic Chorioamnionitis and Fetal Inflammatory Response Syndrome to Increased Risk of Brain Injury in Infants With Preterm Premature Rupture of Membranes. Pediatr. Neurol..

[35] Katz N., Schreiber L., Oron A., Halachmi S., Kohelet D. (2017). Inflammatory Response in Preterm Newborns Born after Prolonged Premature Rupture of Membranes: Is There a Correlation with Placental Histological Findings?. Isr. Med. Assoc. J. IMAJ.

[36] Budal E.B., Kessler J., Eide G.E., Ebbing C., Collett K. (2024). Placental pathology and neonatal morbidity: Exploring the impact of gestational age at birth. BMC Pregnancy Childbirth.

[37] Ramsey P.S., Lieman J.M., Brumfield C.G., Carlo W. (2005). Chorioamnionitis Increases Neonatal Morbidity in Pregnancies Complicated by Preterm Premature Rupture of Membranes. Am. J. Obstet. Gynecol..

[38] Rallis D., Lithoxopoulou M., Pervana S., Karagianni P., Hatziioannidis I., Soubasi V., Tsakalidis C. (2022). Clinical chorioamnionitis and histologic placental inflammation: Association with early-neonatal sepsis. J. Matern. Fetal Neonatal Med..

[39] Oshima Y., Tanaka S., Tsumura K., Tsuda S., So K., Shinagawa T., Yamasaki F., Kawaguchi A., Nomiyama M., Yokoyama M. (2020). Amniotic Fluid Gram Stain and Interleukin-6 Can Predict Early-Onset Neonatal Sepsis. J. Obstet. Gynaecol. Res..

